# Occurrence of *tet*(O/M/O) Mosaic Gene in Tetracycline-Resistant *Campylobacter*

**DOI:** 10.3390/microorganisms8111710

**Published:** 2020-10-31

**Authors:** Lorena Hormeño, Maria J. Campos, Santiago Vadillo, Alberto Quesada

**Affiliations:** 1 Departamento de Bioquímica, Biología Molecular y Genética, Facultad de Veterinaria, Universidad de Extremadura, 10003 Cáceres, Spain; lorena__h@hotmail.com (L.H.); aquesada@unex.es (A.Q.); 2 MARE-Marine and Environmental Sciences Centre, ESTM, Instituto Politécnico de Leiria, 2520-641 Peniche, Portugal; 3 Departamento de Sanidad Animal, Facultad de Veterinaria, Universidad de Extremadura, 10003 Cáceres, Spain; svadillo@unex.es

**Keywords:** *Campylobacter coli*, *Campylobacter jejuni*, tetracycline resistance, *tet* genes, mosaic *tet*(O/M/O)

## Abstract

*Campylobacter* is one of the most important microorganisms responsible for foodborne diseases in the EU. In this study, we investigated resistance to tetracycline in 139 *Campylobacter jejuni* and *Campylobacter coli* samples isolated from human clinical cases. From these, 110 were resistant to tetracycline, with MIC (minimal inhibitory concentration) varying in a range of 1 to >512 μg/mL, and 109 (78.4%) carried *tet*(O), a gene that confers resistance to tetracycline through the expression of a protein that confers protection to the ribosome. Amongst the tetracycline-resistant isolates, one *C. jejuni* (HCC30) was the only *tet(O)*-negative sample, presenting an MIC of 256 μg/mL. Instead, the mosaic gene *tet*(O/M/O) was found in HCC30 and, as far as we know, this is the first description of this chimeric gene originating from homologous recombination between *tet*(O) and *tet*(M). The previously described mosaic gene *tet*(O/32/O), also found in *Campylobacter*, presents a chimeric structure very similar to that of *tet*(O/M/O), affecting domains II and III of encoded proteins distantly related to the elongation factor G (EF-G). The *tet*(O/M/O) mosaic gene has been found in nucleotide databases in several genomes of *Campylobacter* isolated from different origins, indicating its frequent acquisition, even though it can be undetected through screening by PCR with specific *tet*(O) primers. In this work, we address the improvement of classical PCR to efficiently diagnose the most prevalent tetracycline resistance determinants in *Campylobacter*, including *tet*(O/M/O), which should be taken into account in the optimization of campylobacteriosis treatments.

## 1. Introduction

*Campylobacter* spp. infections have been the most dominant gastrointestinal disease reported in the EU since 2005. Generally, this infection is self-limiting but can be the trigger for severe illness, such as Guillain–Barré syndrome and autoimmune inflammatory conditions [1]. The level of antimicrobial resistance of this zoonosis, in European member states, according to the European Committee on Antimicrobial Susceptibility Testing EUCAST ecological cut-off values, varies from 0.5% to gentamicin up to 45.4% to tetracycline, in *Campylobacter jejuni* isolated from human infections, whilst in *Campylobacter coli*, 1.8% were resistant to gentamicin and 68.3% were resistant to tetracycline [1]. In isolates obtained from animals, the same pattern of resistance occurs in both *C. coli* and *C. jejuni* isolated from fattening pigs and calves [1]. The *Campylobacter* genus, being both a zoonotic and enteric microorganism, has acquired several antimicrobial resistances due to its exposure to antimicrobials used in the treatment/prophylaxis of disease in food-producing animals, companion animals and humans [2]. Some of the most used antimicrobials in the treatment of campylobacteriosis are macrolides and fluoroquinolones [3,4], and occasionally aminoglycosides and oral beta-lactams, however, tetracycline, fluoroquinolones, macrolides, florfenicol and trimethoprim–sulfamethoxazole are the drugs against which *Campylobacter* presents the greatest resistance [4].

Tetracyclines are a group of antibiotics extensively used in the treatment of both animal and human infections, but also as prophylaxis agents and as growth promotors in animal husbandry [5]. This antimicrobial presents a broad spectrum of activities and is low cost, making it suitable for incorporation in animal feeds at subtherapeutic doses to act as a growth promotor which can be a practice responsible for the development of bacterial resistance [6,7]. The first tetracycline resistance genes discovered were genes *tet*(A) to *tet*(E), in Gram-negative bacteria, mediating efflux pumps and genes *tet*(L) to *tet*(N), in Gram-positive cocci, conferring resistance by encoding ribosomal protection proteins [8]. *tet*(O) was found in a self-transmissible plasmid from *C. coli* and it was thought to have diverged from the gene *tet*(M) [9]. Today, there are more than 60 tetracycline resistance genes described and besides the already mentioned mechanisms of action, resistance mediated by enzymatic inactivation of the molecule was also reported [7,10,11,12,13]. The improvement of diagnostic techniques and the elimination of false positives corroborate the knowledge that the *tet*(O) sequence is the only resistance determinant to tetracycline in *C. jejuni* and *C. coli* [4,14], especially associated with the acquisitions of plasmids containing the gene [15,16,17], although it can also be found in the chromosome [18,19,20]. Mosaic genes are common amongst genes responsible for ribosomal protection proteins and several of these elements have been described, with the majority deriving from *tet*(O), *tet*(W) and *tet*32, including *tet*(O/32/O) recently found in *C. coli* and *C. jejuni* isolated from humans [7]. 

In this work, we describe the detection of a *tet*(O/M/O) mosaic gene in *C. jejuni*, analyze its relevance for the molecular diagnosis of antimicrobial resistance and discuss implications for structure–function relationships of ribosomal protection factors. 

## 2. Materials and Methods 

### 2.1. Microbial Growth And Antibiotic Resistance Testing

*Campylobacter* spp. of human origin were isolated between 2010 and 2012 (139 strains; 132 *C. jejuni* and seven *C. coli*) and were described previously [21,22,23]. Cultivation procedures of the isolates included culture on blood agar in a microaerophilic atmosphere (CampyGen™; Thermo Scientific, Lenexa, KS, USA) at 42 °C for 24–48 h [21,22]. Tetracycline minimal inhibitory concentrations (MICs) of the isolates were determined by agar dilution methods according to the Clinical and Laboratory Standards Institute CLSI [24].

### 2.2. PCR Screening of Resistance Determinants

The presence of *tet*(O) genes was investigated using PCR with primers and conditions already described. Primers specific for *tet(O)*, tetOF (5’-gcgttttgtttatgtgcg) and tetOR (5’-atggacaacccgacagaag) were modified from primers previously described by Bacon et al. [25] and degenerated primers, designed to amplify all *tet* genes, tetDF (5’-GCTCA(T/C)GTTGA(T/C)GCAGGAA) and tetDR (5’-AGGATTTGGCGG(C/G)ACTTC(G/T)A) [26], were used in PCR with the following conditions: initial melting temperature of 95 °C for 5 min; 30 cycles of 95 °C for 30 s, 55 °C or 50 °C (for specific and degenerated primers, respectively) for 30 s and 72 °C for 1.5 min and a final extension step of 72 °C for 2 min. Reagents used in the PCR reactions contained 0.2 mmol/L dNTPs (Takara-Clontech, Kusatsu, Shiga, Japan), 0.5 μmol/L of each primer, 0.025 U/μL Taq polymerase (Biotools, Madrid, Spain), 1 × PCR buffer with 1.5 mmol/L MgCl_2_ (Biotools), and 5 μL of DNA template in a total volume of 50 μL. PCR products (558 or 1293 bp, for specific or degenerated primers, respectively) were purified with a Speedtools PCR clean-up kit (Biotools, Madrid, Spain) according to the manufacturer’s instructions and Sanger sequencing was performed by the facilities of the Universidad de Extremadura, Spain (STAB).

### 2.3. Bioinformatic Tools

Comparison of DNA and protein sequences was performed by the basic local alignment search tool (BLAST) using the nucleotide collection nr/nt or the non-redundant protein sequence (nr) databases [27]. Clustal X 2.0 software was used for multiple sequence alignments. 

## 3. Results

Seventy-nine percent of the tested *Campylobacter* isolates (104 *C. jejuni* and six *C. coli* out of 139, Supplementary Table S1), were resistant to tetracycline according to the CLSI [24] breakpoint (MIC ≥ 16 mg/L) and the presence of the *tet*(O) gene, determined by PCR, almost matched the resistance phenotype of the isolates with the unique exception of *C. jejuni* HCC30, which presented a tetracycline MIC of 256 mg/L (Table 1). Thus, among the analyzed isolates, the *tet*(O) gene was the main resistance determinant carried by 99.1% (109/110) of tetracycline-resistant isolates.

**Figure 1:**
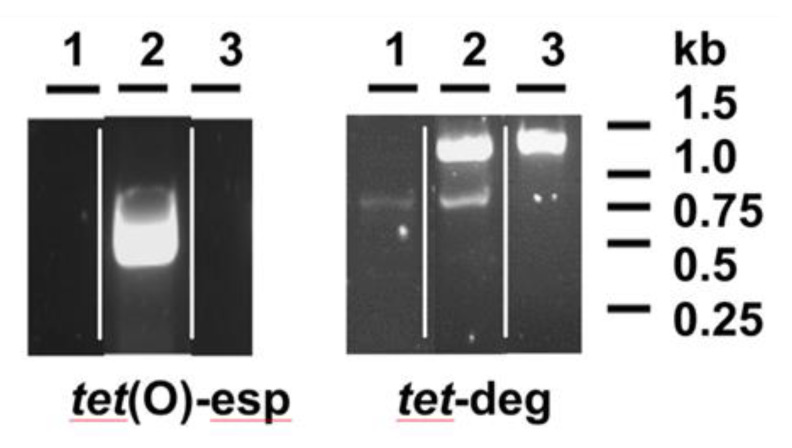
PCR detection of *tet*(O) sequences from *Campylobacter*. DNA samples were amplified by using specific primers (tet(O)-esp) or degenerated primers (tet-deg), according to conditions explained in the Material and Methods section and analyzed by agarose gel electrophoresis. Strains analyzed are: 1, HCC69 (susceptible to tetracycline); 2, HSA1 (resistant to tetracycline); 3, HCC30 (resistant to tetracycline). The figure presented is composed of images obtained from different gels.

The specific primers used in this work to detect the *tet*(O) gene [25] had been extensively and successfully used [19] and amplified the expected 0.5 Kb DNA fragment in most tetracycline-resistant isolates as HSA1 (Figure 1). The same was not possible with isolate HCC30, for which PCR fragment amplification failed, in a similar way to the susceptible background of isolate HCC69 (Figure 1). However, degenerated primers, designed based on available sequences for ribosomal protection proteins [26], made possible the production of a PCR fragment with the expected size (1 to 1.5 Kb) in both tetracycline-resistant isolates, HCC30 and HSA1, but not in HCC69 (Figure 1). The DNA fragment amplified in HCC30 proved to be 100% identical to three sequences present in *C. coli* (accession no. AY394560.1 and MF134831) and *C. jejuni* (CP023446), and closely related (presenting up to three single nucleotide polymorphisms) to nine sequences from *C. jejuni* (CP048769.1, CP048767.1, CP048765.1, CP059964.1, CP048764.1, CP048763.1, CP048756.1, CP048771.1, KF864551.1), five from *C. coli* (MF037584.1, KC876752.1, KC876751.1, KC876749.1, CP044164.1) and one from *C. fetus* (CP027287.1), all of them annotated as *tetO*. 

**Figure 2:**
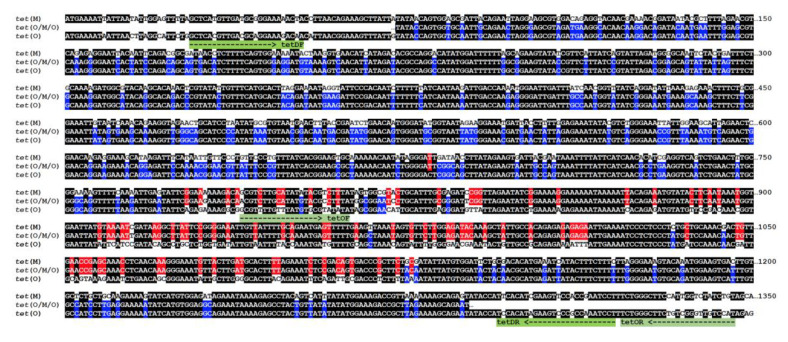
Chimeric structure of *tet*(O/M/O) mosaic gene. Nucleotide sequences shown are: *tet*(M), X04388; *tet*(O), CP044175 and *tet*(O/M/O), this work (100% identical to AY394560.1). Identity color code: black, homology between three sequences; blue, homology between *tet*(O) and *tet*(O/M/O); red, homology between *tet*(M) and *tet*(O/M/O). Primers used in this work are shown below the sequences and indicated in green.

**Figure 3:**
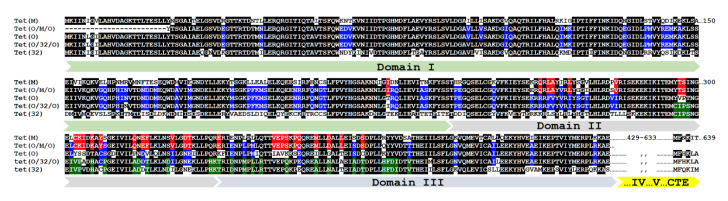
Chimeric structure of Tet(O/M/O) and Tet(O/32/O) mosaic proteins. Amino acid sequence shown are: Tet(M), WP_004632336; Tet(O), EAI7795628; Tet(32), WP_002602099.1; Tet(O/32/O), AINH01000038; Tet(O/M/O), this work (100% identical to WP_002872163, encoded by AY394560.1). Identity color code: black, homology ≥ 80% among sequences; blue, homology ≥ 60% among *tet*(O), *tet*(O/M/O) and/or Tet(O/32/O); red, *tet*(M) and *tet*(O/M/O); green, *tet*(32) and *tet*(O/32/O).

Analysis of these sequences revealed it to correspond to a not yet described class of tetracycline-resistant gene that presents the replacement of the 776–1127 bp internal fragment of *tet*(O) by the homologous sequence from *tet*(M), a new mosaic structure that will be named hereafter as *tet*(O/M/O) (Figure 2). Strains of *C. jejuni* and *C. coli* are known to present the mosaic *tet*(O/32/O) [7] and, as far as we know, this is the first report describing the existence of the *tet*(O/M/O) mosaic sequence mobilized among different species of *Campylobacter*, although a recent report mentioned its occurrence in particular sequence types of *C. jejuni* [28]. Interestingly, both *tet*(O/32/O) and *tet*(O/M/O) mosaic genes from *Campylobacter* share the same chimeric structure, with a DNA fragment of similar length (about 300 bp) from *tet*(32) or *tet*(M) inserted in nearly the same position of *tet*(O). Encoded proteins are thus chimeras with TetM or Tet32 insertions with corresponding polymorphisms mapped to structural domains II and III of the ribosomal protection proteins (Figure 3) and their distantly related homolog, translation elongation factor G (EF-G) from which Tet proteins have obtained the capacity to bind to the ribosome and release the tetracycline molecule [29], resulting in resistance to this antimicrobial.

## 4. Discussion

The percentage of resistance to tetracycline showed by the isolates studied was much higher compared to the resistance found in other works with *Campylobacter* spp. isolated from humans. Elhadidy et al [30], found 49.7% resistance to tetracycline, much in accordance with the level of resistance found by Marotta et al. [31], with 49.0% resistant isolates, even though this last study used the epidemiological cut-off values (ECOFFs) defined by EUCAST [32]. Considering the same resistance definition, the study carried out in Spain by Ocejo et al. [33] showed that the level of resistance in *Campylobacter* spp., isolated from animals, was 76.5%. Tetracyclines were extensively used in Spain in animal husbandry, being 40% of all the antibiotics consumed in 2013 [34] and the high results of resistance found in our work might reflect this practice.

The use of degenerated primers for the amplification of *tet* genes originated the amplification of a fragment already present in nucleotide databases, which was described in a wide spectrum of *Campylobacter* species and origins, such as *C. coli* isolated from humans, turkeys and pigs, *C. fetus* isolated from humans and *C. jejuni* isolated from chickens. Analysis of the sequence revealed it to correspond to a not yet described class of tetracycline-resistant gene that presents a mosaic structure with the replacement of the 776-1127 bp internal fragment of *tet*(O) by the homologous sequence from *tet*(M) (Figure 2). This *tet*(O/M/O) mosaic gene might have evolved from a homologous recombination event between *tet*(O) and *tet*(M), encoding a ribosomal protection factor widely spread in Firmicutes from the Bacilli class, such as enterococci and streptococci [26,27,35,36], many of which share the mammalian gastrointestinal tract environment with *Campylobacter*, which, together with *Megasphaera* and *Riemerella,* are the only three Gram-negative genera in which *tet* mosaic genes have been reported [13]. Curiously, all these genera are common inhabitants of the gastrointestinal tract of both animals and humans. 

*tet*(O/W/O) mosaic genes were the first reported in [37] and since then several others, including different chimeras between *tet*(M), *tet*(O), *tet*(S), *tet*(W) and *tet*(32), have been described and proved to be functional with resistance levels comparable to the non-mosaic genes [7], also including *tet*(O/M/O), since plasmid pCC31 carrying an identical sequence to that found in HCC30 mobilized tetracycline resistance among *Campylobacter* strains [38]. Taking into account that the National Center of Biotechnology Information (NCBI) nucleotide collection (nr/nt) database presents 126 full coding sequences for *tet*(O) genes from *Campylobacter*, among which 17 and five correspond to the *tet*(O/M/O) and *tet*(O/32/O) mosaic genes, respectively (data available on 21st October 2020), and that both chimeras present similar domain organization, makes it advisable to use the degenerated primers previously described [26] in PCR for an accurate identification of tetracycline-resistant determinants of strains producing negative results in conventional PCR by specific primers.

The mechanism of action of tetracycline is based on the binding of the molecule at the A site of the 30S subunit of the ribosome, a connection that involves the 16S rRNA [39]. The presence of tetracycline blocks tRNA binding and consequently the inhibition of protein synthesis [40]. *tet*(O) and *tet*(M) are paralogs of EF-G, a translation GTPase, and are able to remove tetracycline from the ribosome dependent on the GTP hydrolysis [41]. There is not much available information about the functional role of domains II and III of EF-G, since ribosomal binding determinants are located in domain IV and CTE, the C-terminal extension that is lacking in EF-G, whereas GTPase activity centers are in domain I, the G-domain [29]. The fact that, during evolution of ribosomal protection proteins, in *Campylobacter*, all *tet*(O) mosaic sequences present insertions of genes *tet*(32) and *tet*(M) in regions encoding domains II and III (Figure 2 and Figure 3), strongly suggests that still unknown structural and/or functional determinants might have driven the selection of these chimeric sequences, under exposure to the antibiotic used for infection treatment in humans or prophylaxis/growth enhancement in animals [42]. 

## 5. Conclusions

This work describes the identification of a new class of tetracycline-resistant determinants in *Campylobacter*, the *tet*(O/M/O) mosaic gene. The occurrence of this genetic element suggests that recombination exchange could have taken place within the same bacterium carrying co-existing *tet*(O) and *tet*(M) resistance determinants. Whether this mosaic gene was transferred to *Campylobacter* by means of a plasmid, by conjugative transposons, or if recombination occurred with a resident *tet*(O) gene after natural transformation of *Campylobacter,* remains unknown. The relevant fact is that the co-existence of closely related sequences in the same environment might provide bacteria with genetic tools to accelerate the evolution of antimicrobial resistance determinants. Thus, besides recognizing the *tet*(O/M/O) mosaic gene for the first time, this work provides a new PCR strategy to detect this resistance determinant, preventing it from occurring as a false negative, a finding that might be relevant for clinicians and/or biochemists interested in diagnosing antimicrobial resistance and/or understanding the evolution of ribosomal protection proteins.

## Figures and Tables

**Table 1:** Involvement of *tet*(O) in tetracycline resistance.

		MIC ^#^ (mg/L)								
Species	Tet(O) *	≤4	8	16	32	64	128	256	≥512	Sum
***C. jejuni***	+	0	0	3	3	24	50	9	14	103
	-	26	2	0	0	0	0	0	1	29
***C. coli***	+	0	0	0	1	1	0	3	1	6
	-	1	0	0	0	0	0	0	0	1

^#^ Minimal inhibitory concentration. * Presence or absence of *tet*(O) as revealed by PCR with specific primers [25]. Degenerated primers [26] were used to screen, by PCR, all strains lacking *tet*(O), among which only HCC30 (Table S1) was positive.

## Supplementary Materials

The following are available online at https://www.mdpi.com/2076-2607/8/11/1710/s1, Table S1: Tetracycline phenotype and genotype of *Campylobacter* isolates.

## References

[1] European Food Safety Authority, European Centre for Disease Prevention and Control (2019). The European Union summary report on antimicrobial resistance in zoonotic and indicator bacteria from humans, animals and food in 2017. EFSA J..

[2] Dai L., Sahin O., Grover M., Zhang Q. (2020). New and alternative strategies for the prevention, control, and treatment of antibiotic-resistant *Campylobacter*. Transl. Res..

[3] Arakawa Y. (2020). Systematic research to overcome newly emerged multidrug-resistant bacteria. Microbiol. Immunol..

[4] Tang Y., Fang L., Xu C., Zhang Q. (2017). Antibiotic resistance trends and mechanisms in the foodborne pathogen, *Campylobacter*. Anim. Health Res. Rev..

[5] Chopra I., Roberts M. (2001). Tetracycline antibiotics: Mode of action, applications, molecular biology, and epidemiology of bacterial resistance. Microbiol. Mol. Biol. Rev..

[6] Granados-Chinchilla F., Rodríguez C. (2017). Tetracyclines in Food and Feedingstuffs: From Regulation to Analytical Methods, Bacterial Resistance, and Environmental and Health Implications. J. Anal. Methods Chem..

[7] Warburton P.J., Amodeo N., Roberts A.P. (2016). Mosaic tetracycline resistance genes encoding ribosomal protection proteins. J. Antimicrob. Chemother..

[8] Zilhao R., Papadopoulou B., Courvalin P. (1988). Occurrence of the *Campylobacter* resistance gene *tetO* in *Enterococcus* and *Streptococcus* spp.. Antimicrob. Agents Chemother..

[9] Sougakoff W., Papadopoulou B., Nordmann P., Courvalin P. (1987). Nucleotide sequence and distribution of gene *tetO* encoding tetracycline resistance in *Campylobacter coli*. FEMS Microbiol. Lett..

[10] Leski T.A., Bangura U., Jimmy D.H., Ansumana R., Lizewski S.E., Stenger D.A., Taitt C.R., Vora G.J. (2013). Multidrug-resistant *tet*(X)-containing hospital isolates in Sierra Leone. Int. J. Antimicrob. Agents.

[11] Marosevic D., Kaevska M., Jaglic Z. (2017). Resistance to the tetracyclines and macrolide-lincosamide-streptogramin group of antibiotics and its genetic linkage—A review. Ann. Agric. Environ. Med..

[12] Park J., Gasparrini A., Reck M.R., Symister C.T., Elliott J.L., Vogel J.P., Wencewicz T.A., Dantas G., Tolia N.H.M. (2017). Plasticity, dynamics, and inhibition of emerging tetracycline resistance enzymes. Nat. Chem. Biol..

[13] Marilyn C. Roberts. http://faculty.washington.edu/marilynr/.

[14] Lynch C., Hawkins K., Lynch H., Egan J., Bolton D., Coffey A., Lucey B. (2019). Investigation of molecular mechanisms underlying tetracycline resistance in thermophilic *Campylobacter* spp. suggests that previous reports of *tet*(A)-mediated resistance in these bacteria are premature. Gut Pathog..

[15] Fabre A., Oleastro M., Nunes A., Santos A., Sifré E., Ducournau A., Bénéjat L., Buissonnière A., Floch P., Mégraud F. (2018). Whole-Genome Sequence Analysis of Multidrug-Resistant *Campylobacter* Isolates: A Focus on Aminoglycoside Resistance Determinants. J. Clin. Microbiol..

[16] French N.P., Zhang J., Carter G.P., Midwinter A.C., Biggs P.J., Dyet K., Gilpin B.J., Ingle D.J., Mulqueen K., Rogers L.E. (2019). Genomic Analysis of Fluoroquinolone- and Tetracycline-Resistant *Campylobacter jejuni* Sequence Type 6964 in Humans and Poultry, New Zealand, 2014–2016. Emerg. Infect. Dis..

[17] Tenover F.C., Williams S., Gordon K.P., Nolan C., Plorde J.J. (1985). Survey of plasmids and resistance factors in *Campylobacter jejuni* and *Campylobacter coli*. Antimicrob. Agents Chemother..

[18] Crespo M.D., Olson J.W., Altermann E., Siletzky R.M., Kathariou S. (2012). Chromosomal *tet*(O)-harboring regions in *Campylobacter coli* isolates from turkeys and swine. Appl. Environ. Microbiol..

[19] Pratt A., Korolik V. (2005). Tetracycline resistance of Australian *Campylobacter jejuni* and *Campylobacter coli* isolates. J. Antimicrob. Chemother..

[20] Tang Y., Meinersmann R.J., Sahin O., Wu Z., Dai L., Carlson J., Plumblee Lawrence J., Genzlinger L., LeJeune J.T., Zhang Q. (2017). Wide but Variable Distribution of a Hypervirulent *Campylobacter jejuni* Clone in Beef and Dairy Cattle in the United States. Appl. Environ. Microbiol..

[21] Hormeño L., Palomo G., Ugarte-Ruiz M., Porrero M.C., Borge C., Vadillo S., Píriz S., Domínguez L., Campos M.J., Quesada A. (2016). Identification of the main quinolone resistance determinant in *Campylobacter jejuni* and *Campylobacter coli* by MAMA-DEG PCR. Diagn. Microbiol. Infect. Dis..

[22] Hormeño L., Ugarte-Ruiz M., Palomo G., Florez-Cuadrado D., Vadillo S., Píriz S., Domínguez L., Campos M.J., Quesada A. (2018). *ant(6)-I* Genes Encoding Aminoglycoside O-Nucleotidyltransferases Are Widely Spread Among Streptomycin Resistant Strains of *Campylobacter jejuni* and *Campylobacter coli*. Front. Microbiol..

[23] Mourkas E., Florez-Cuadrado D., Pascoe B., Calland J.K., Bayliss S.C., Mageiros L., Méric G., Hitchings M.D., Quesada A., Porrero C. (2019). Gene pool transmission of multidrug resistance among *Campylobacter* from livestock, sewage and human disease. Environ. Microbiol..

[24] Clinical and Laboratory Standard Institute (CLSI) (2016). Methods for Antimicrobial Dilution and Disk Susceptibility Testing of Infrequently Isolated or Fastidious Bacteria.

[25] Bacon D.J., Alm R.A., Burr D.H., Hu L., Kopecko D.J., Ewing C.P., Trust T.J., Guerry P. (2000). Involvement of a plasmid in virulence of *Campylobacter jejuni* 81-176. Infect. Immun..

[26] Barbosa T.M., Scott K.P., Flint H.J. (1999). Evidence for recent intergeneric transfer of a new tetracycline resistance gene, *tet*(W), isolated from *Butyrivibrio fibrisolvens*, and the occurrence of *tet(O)* in ruminal bacteria. Environ. Microbiol..

[27] National Center for Biotechnology Information U.S. National Library of Medicine 8600 Rockville Pike, Bethesda MD, 20894 USA.

[28] Lopes B.S., Strachan N., Ramjee M., Thomson A., MacRae M., Shaw S., Forbes K.J. (2019). Nationwide Stepwise Emergence and Evolution of Multidrug-Resistant *Campylobacter jejuni* Sequence Type 5136, United Kingdom. Emerg. Infect. Dis..

[29] Dönhöfer A., Franckenberg S., Wickles S., Berninghausen O., Beckmann R., Wilson D.N. (2012). Structural basis for TetM-mediated tetracycline resistance. Proc. Natl. Acad. Sci. USA.

[30] Elhadidy M., Ali M.M., El-Shibiny A., Miller W.G., Elkhatib W.F., Botteldoorn N., Dierick K. (2020). Antimicrobial resistance patterns and molecular resistance markers of *Campylobacter jejuni* isolates from human diarrheal cases. PLoS ONE.

[31] Marotta F., Garofolo G., di Marcantonio L., di Serafino G., Neri D., Romantini R., Sacchini L., Alessiani A., Di Donato G., Nuvoloni R. (2019). Antimicrobial resistance genotypes and phenotypes of *Campylobacter jejuni* isolated in Italy from humans, birds from wild and urban habitats, and poultry. PLoS ONE.

[32] European Committee on Antimicrobial Susceptibility Testing. https://eucast.org.

[33] Ocejo M., Oporto B., Hurtado A. (2019). Occurrence of *Campylobacter jejuni* and *Campylobacter coli* in Cattle and Sheep in Northern Spain and Changes in Antimicrobial Resistance in Two Studies 10-years Apart. Pathogens.

[34] European Medicines Agency (EMA) (2018). European Surveillance of Veterinary Antimicrobial Consumption. Sales of Veterinary Antimicrobial Agents in 30 European Countries in 2015 (EMA/387934/2015).

[35] Marasini D., Karki A.B., Buchheim M.A., Fakhr M.K. (2018). Phylogenetic Relatedness Among Plasmids Harbored by *Campylobacter jejuni and Campylobacter coli* Isolated From Retail Meats. Front. Microbiol..

[36] Spigaglia P., Barbanti F., Mastrantonio P. (2008). Tetracycline resistance gene *tet*(W) in the pathogenic bacterium *Clostridium difficile*. Antimicrob. Agents Chemother..

[37] Stanton T.B., Humphrey S.B. (2003). Isolation of tetracycline-resistant Megasphaera elsdenii strains with novel mosaic gene combinations of *tet*(O) and *tet*(W) from swine. Appl. Environ. Microbiol..

[38] Batchelor R.A., Pearson B.M., Friis L.M., Guerry P., Wells J.M. (2004). Nucleotide sequences and comparison of two large conjugative plasmids from different *Campylobacter* species. Microbiology.

[39] Speer B.S., Shoemaker N.B., Salyers A.A. (1992). Bacterial resistance to tetracycline: Mechanisms, transfer, and clinical significance. Clin. Microbiol. Rev..

[40] Yu A.M., Choi Y.H., Tu M.J. (2020). RNA Drugs and RNA Targets for Small Molecules: Principles, Progress, and Challenges. Pharmacol. Rev..

[41] Li W., Atkinson G.C., Thakor N.S., Allas U., Lu C.C., Chan K.Y., Tenson T., Schulten K., Wilson K.S., Hauryliuk V. (2013). Mechanism of tetracycline resistance by ribosomal protection protein Tet(O). Nat. Commun..

[42] Thaker M., Spanogiannopoulos P., Wright G.D. (2010). The tetracycline resistome. Cell. Mol. Life Sci..

