# Supplementary material for: Occurrence of tet(O/M/O) Mosaic Gene in Tetracycline-Resistant Campylobacter
Source: Microorganisms. 2020 Oct 31;8(11):1710. doi: 10.3390/microorganisms8111710 (PMC7693602; doi:10.3390/microorganisms8111710)
Supplement: Supplementary file 1 [file microorganisms-08-01710-s001.pdf]

Table S1 (supplementary). Tetracycline phenotype and genotype of *Campylobacter* isolates.

**Name[1]**	**Species**	**CMI-Tet (mg/L)**	***tet(O)***
**HCC1**	*C. jejuni*	32	+
**HCC2**	*C. jejuni*	128	+
**HCC3**	*C. jejuni*	128	+
**HCC4**	*C. jejuni*	>512	+
**HCC5**	*C. jejuni*	256	+
**HCC6**	*C. jejuni*	128	+
**HCC7**	*C. jejuni*	128	+
**HCC8**	*C. jejuni*	64	+
**HCC9**	*C. jejuni*	64	+
**HCC10**	*C. jejuni*	2	-
**HCC11**	*C. jejuni*	128	+
**HCC13**	*C. jejuni*	128	+
**HCC14**	*C. jejuni*	128	+
**HCC15**	*C. jejuni*	256	+
**HCC16**	*C. jejuni*	4	-
**HCC17**	*C. jejuni*	128	+
**HCC18**	*C. jejuni*	4	-
**HCC19**	*C. jejuni*	128	+
**HCC20**	*C. jejuni*	>512	+
**HCC21**	*C. jejuni*	64	+
**HCC22**	*C. jejuni*	128	+
**HCC23**	*C. jejuni*	>512	+
**HCC24**	*C. jejuni*	128	+
**HCC25**	*C. jejuni*	128	+
**HCC26**	*C. jejuni*	1	-
**HCC27**	*C. jejuni*	2	-
**HCC28**	*C. jejuni*	4	-
**HCC29**	*C. jejuni*	256	+
**HCC30**	*C. jejuni*	256	-
**HCC31**	*C. jejuni*	4	-
**HCC32**	*C. jejuni*	8	-
**HCC33**	*C. jejuni*	64	+
**HCC34**	*C. jejuni*	4	-
**HCC35**	*C. jejuni*	1	-
**HCC36**	*C. jejuni*	4	-
**HCC37**	*C. jejuni*	128	+
**HCC38**	*C. jejuni*	64	+
**HCC40**	*C. jejuni*	64	+
**HCC41**	*C. jejuni*	64	+
**HCC43**	*C. coli*	512	+

**Name**[1]	**Species**	**CMI-Tet (mg/L)**	***tetO***
**HCC44**	*C. jejuni*	0.5	-
**HCC45**	*C. jejuni*	128	+
**HCC46**	*C. jejuni*	16	+
**HCC47**	*C. jejuni*	128	+
**HCC48**	*C. jejuni*	512	+
**HCC49**	*C. jejuni*	128	+
**HCC50**	*C. jejuni*	512	+
**HCC51**	*C. jejuni*	>512	+
**HCC52**	*C. jejuni*	128	+
**HCC54**	*C. jejuni*	128	+
**HCC55**	*C. jejuni*	512	+
**HCC56**	*C. jejuni*	>512	+
**HCC57**	*C. jejuni*	2	-
**HCC58**	*C. jejuni*	128	+
**HCC59**	*C. jejuni*	512	+
**HCC60**	*C. jejuni*	512	+
**HCC61**	*C. jejuni*	16	+
**HCC62**	*C. jejuni*	4	-
**HCC63**	*C. jejuni*	512	+
**HCC64**	*C. jejuni*	128	+
**HCC65**	*C. jejuni*	256	+
**HCC66**	*C. jejuni*	32	+
**HCC67**	*C. jejuni*	256	+
**HCC68**	*C. jejuni*	128	+
**HCC69**	*C. jejuni*	4	-
**HCC70**	*C. jejuni*	64	+
**HCC71**	*C. jejuni*	64	+
**HCC72**	*C. jejuni*	128	+
**HCC73**	*C. jejuni*	64	+
**HCC74**	*C. jejuni*	64	+
**HCC75**	*C. jejuni*	128	+
**HCC76**	*C. jejuni*	128	+
**HCC77**	*C. jejuni*	512	+
**HCC81**	*C. jejuni*	128	+
**HCC82**	*C. jejuni*	128	+
**HCC83**	*C. jejuni*	128	+
**HCC86**	*C. jejuni*	128	+
**HCC87**	*C. jejuni*	2	-
**HCC88**	*C. jejuni*	64	+
**HCC89**	*C. jejuni*	64	+
**HCC90**	*C. jejuni*	128	+

Name[1]	Species	CMI-Tet (mg/L)	*tetO*
**HCC91**	*C. jejuni*	64	+
**HCC92**	*C. jejuni*	64	+
**HCC95**	*C. jejuni*	64	+
**HSA 1**	*C. jejuni*	64	+
**HSA 2**	*C. jejuni*	128	+
**HSA4**	*C. jejuni*	64	+
**HSA5**	*C. jejuni*	256	+
**HSA6**	*C. jejuni*	64	+
**HSA7**	*C. jejuni*	4	-
**HSA8**	*C. jejuni*	64	+
**HSA9**	*C. jejuni*	64	+
**HSA10**	*C. jejuni*	128	+
**HSA11**	*C. jejuni*	128	+
**HSA12**	*C. jejuni*	1	-
**HSA13**	*C. jejuni*	128	+
**HSA14**	*C. jejuni*	4	-
**HSA15**	*C. jejuni*	1	-
**HSA16**	*C. coli*	32	+
**HSA17**	*C. jejuni*	32	+
**HSA18**	*C. jejuni*	1	-
**HSA19**	*C. jejuni*	1	-
**HSA20**	*C. coli*	64	+
**HSA21**	*C. jejuni*	128	+
**HSA22**	*C. jejuni*	128	+
**HSA23**	*C. jejuni*	256	+
**HSA24**	*C. jejuni*	128	+
**HSA25**	*C. jejuni*	64	+
**HSA26**	*C. jejuni*	64	+
**HSA27**	*C. jejuni*	128	+
**HSA28**	*C. coli*	256	+
**HSA29**	*C. jejuni*	128	+
**HSA30**	*C. jejuni*	128	+
**HSA31**	*C. jejuni*	1	-
**HSA32**	*C. jejuni*	128	+
**HSA33**	*C. jejuni*	64	+
**HSA35**	*C. jejuni*	128	+
**HSA36**	*C. jejuni*	64	+
**HSA37**	*C. jejuni*	128	+
**HSA38**	*C. jejuni*	>512	+
**HSA39**	*C. jejuni*	16	+
**HSA40**	*C. jejuni*	128	+

**Name[1]**	**Species**	**CMI-Tet (mg/L)**	***tetO***
**HSA41**	*C. jejuni*	4	-
**HSA42**	*C. jejuni*	128	+
**HSA43**	*C. jejuni*	128	+
**HSA44**	*C. jejuni*	64	+
**HSA45**	*C. jejuni*	128	+
**HSA46**	*C. coli*	256	+
**HSA47**	*C. jejuni*	4	-
**HSA48**	*C. jejuni*	128	+
**HNA1**	*C. coli*	1	-
**HNA2**	*C. jejuni*	4	-
**HNA3**	*C. jejuni*	128	+
**HNA4**	*C. coli*	256	+
**HNA5**	*C. jejuni*	2	-
**HNA6**	*C. jejuni*	512	+
**HNA7**	*C. jejuni*	8	-
**HNA8**	*C. jejuni*	128	+
**HNA9**	*C. jejuni*	512	+

[1]*St*rains analyzed in this work have been previously and partially characterized (Hormeño et al., 2016; Hormeño et al., 2018; Mourkas et al., 2019).
